# Multiple Severe Intracranial Stenoses with Ischemic Stroke in Neuroborreliosis-associated Cerebral Vasculitis: Endovascular Treatment Strategies and Literature Review

**DOI:** 10.1007/s00062-024-01447-7

**Published:** 2024-08-12

**Authors:** Kamran Hajiyev, Michael Forsting, Alexandru Cimpoca, Ali Khanafer, Hansjörg Bäzner, Hans Henkes

**Affiliations:** 1 Neuroradiologische Klinik, Klinikum Stuttgart, 70174 Stuttgart, Germany; 2 Neurologische Klinik, Klinikum Stuttgart, 70174 Stuttgart, Germany; 3 Institut für Diagnostische und Interventionelle Radiologie und Neuroradiologie, Universitätsklinikum Essen, 45147 Essen, Germany

**Keywords:** Lyme neuroborreliosis, Cerebral vasculitis, Ischemic stroke, Endovascular treatment, Intracranial stenting, Vessel wall imaging

## Abstract

**Introduction:**

Neuroborreliosis is the disseminated form of Lyme borreliosis and refers to the involvement of the central nervous system by *Borrelia burgdorferi sensu lato* spirochetes. Several reports suggest its emergence as a potential cause of cerebral vasculitis and stroke in children and young adults. The objective of this paper is to highlight endovascular treatment options within this context.

**Methods:**

The medicinal and endovascular treatments of three patients—two adults and one child—with ischemic stroke resulting from neuroborreliosis-associated severe cerebral vasculitis were retrospectively assessed. Detailed descriptions of the clinical course, treatments, and follow-up data for each patient are provided. Additionally, a literature review focusing on endovascular treatment options within this topic was conducted.

**Results:**

Both endovascular and medicinal treatments resulted in excellent clinical outcomes in all three patients, with no observed periprocedural complications. Significant clinical improvement was noted during mid-term follow-up. Follow-up angiographies confirmed stent patency.

***Conclusion*:**

Endovascular interventions as a bailout strategy may enhance clinical outcomes in patients with vascular complications of neuroborreliosis, especially when medicinal therapy alone fails to achieve further improvement. In the setting of severe ischemic stroke with sub-occlusive large vessel stenosis or occlusion, the cause of which is often unknown, it should be considered to prioritize prompt endovascular treatment, even if neuroborreliosis is suspected on admission.

## Introduction

Lyme neuroborreliosis (LNB) is the neurological manifestation of systemic infection with the complex spirochaete *Borrelia burgdorferi*. LNB is reported in approximately 10% of patients with Lyme disease [1]. Neurological manifestations in both early and late stages of neuroborreliosis have been documented mainly in European adults [2]. The clinical presentation can range from acute focal neurological deficits to, more commonly, prodromal symptoms developing over weeks to months and related to the different spirochete species and the age of the patients [3, 4].

In some rare cases, neuroborreliosis may induce inflammation in the cerebral blood vessels, possibly leading to intracranial stenoses, transient ischemic attacks, ischemic strokes, or intracranial hemorrhages [5, 6]. In addition to guideline-based therapy [7], endovascular interventions as a bailout strategy may improve clinical outcomes in patients with severe symptomatic intracranial stenoses, especially when medicinal therapy alone has reached its limits.

## Methods

We present a retrospective analysis of three patients—two adults and one child—with ischemic stroke caused by Lyme neuroborreliosis and cerebral vasculitis who were successfully treated with medicinal and endovascular treatments. Patients admitted to our clinic exhibiting clinical deterioration and new neurological symptoms. Their hospital course, including clinical examinations, laboratory tests, brain imaging as well as treatment protocols, were thoroughly evaluated. Each patient’s clinical progression is individually detailed, supplemented by additional information from follow-up clinical assessments. Furthermore, we conducted a literature review focusing on endovascular treatment for LNB-associated symptomatic cerebral vasculitis within this context.

## Results

### Patient 1

**Fig. 1:**
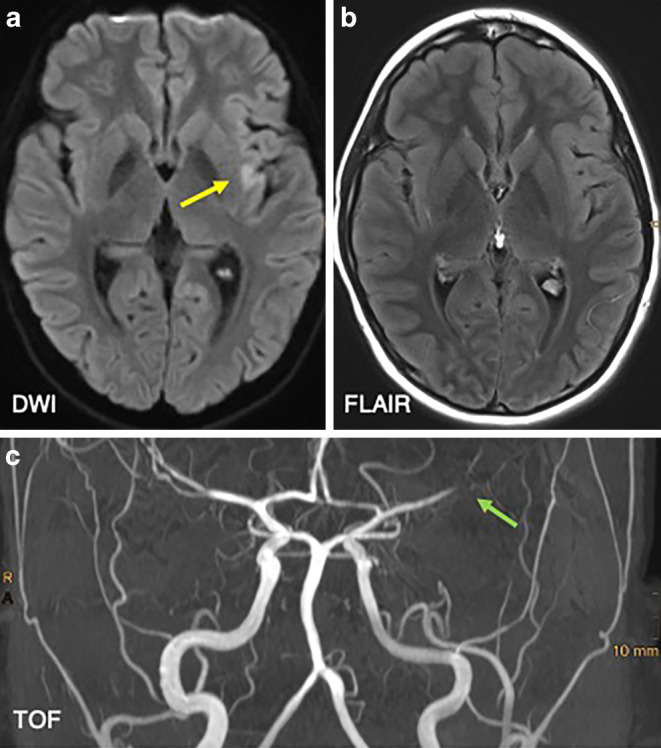
MRI scan of a 13-year-old boy presenting with headache, right arm paresis, and facial palsy. MRI with diffusion-weighted imaging (DWI) (b = 1000 s/mm2, axial view; **(a)** and FLAIR (axial view; **b**) demonstrated a lesion with restricted diffusion of the left insular cortex (yellow arrow) without correlate on FLAIR images. Maximum intensity projection (MIP) images from TOF angiography (**c**) showed a left distal M1 stenosis (green arrow) with flow restriction

A 13-year-old boy presented to the emergency department with severe headache, lightheadedness, and syncopal episodes over a 5-hour period. He was disoriented and extremely agitated in the emergency room. Immediate neurologic examination revealed mild paresis of the right arm, right facial palsy, and meningeal signs. A more detailed history revealed a history of intermittent mild headaches for several weeks. There was no history of tick bites, skin abnormalities, or joint complaints. Consequently, the patient was intubated and transferred for a magnet resonance imaging (MRI) scan. Cranial MRI showed an area of restricted diffusion in the left insular cortex without hyperintense signal changes on fluid attenuated inversion recovery (FLAIR) images. Time-of-flight angiography (TOF) showed loss of arterial flow signal in the left distal M1 segment (Fig. 1). At the time of the MRI examination, there was no definite indication of the cause of the distal luminal narrowing of the left M1 segment. A non-occlusive thrombus, a dissection or an inflammation-induced stenosis of the left middle cerebral artery (MCA) appeared equally possible. Intravenous (IV) contrast was deliberately withheld, and the patient was transferred to the neuroangiography suite.

**Fig. 2:**
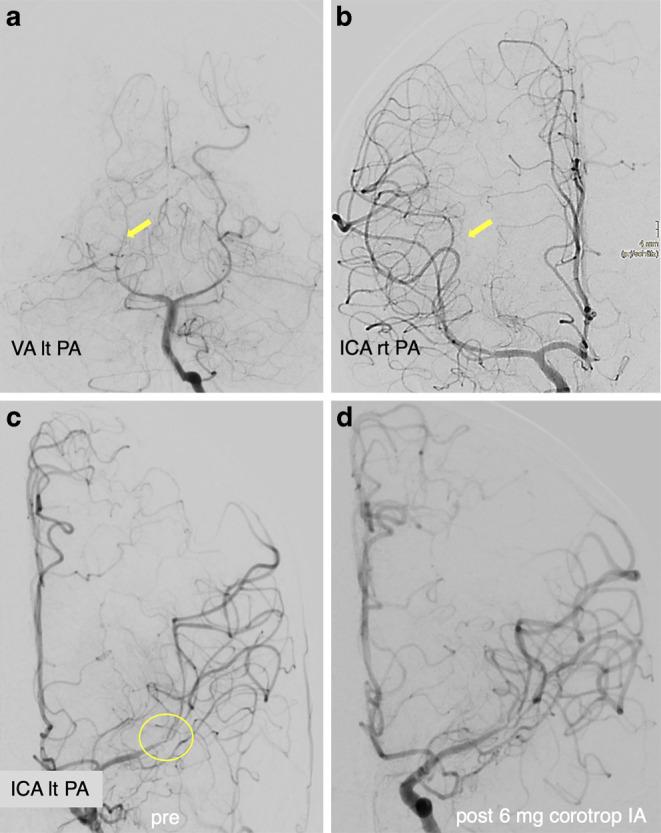
DSA of a 13-year-old boy with symptomatic stenosis of the left MCA. Angiograms of the left vertebral artery (VA) (PA view, **a**) and the right ICA (PA view, **b**) showed no focal stenosis but a generalized narrowing of several pial arteries (e.g., right posterior cerebral artery (PCA), yellow arrow, **a**; right MCA, yellow arrow, **b**). Angiograms of the left ICA showed a significant narrowing of the left distal M1 segment with involvement of the proximal M2 segments (PA view, yellow circle, **c**). A final DSA run of the left ICA after intra-arterial (IA) injection of 6 mg milrinone confirmed an increased diameter of the stenotic left M1/M2 junction and a general dilatation of the left anterior cerebral artery (ACA) and MCA (PA view, **d**)

Digital subtraction angiography (DSA) was performed with the intention of understanding the nature of the narrowing of the left MCA and, if necessary, eliminating it endovascularly. Angiograms of the left internal carotid artery (ICA) showed significant stenosis without thrombus of the left MCA bifurcation involving the distal M1 segment and both proximal M2 segments with perfusion delay in the distal vasculature. No hemodynamically significant focal stenosis was observed in the posterior and right anterior circulation. However, the caliber of all cerebral arteries appeared to be reduced (Fig. 2).

These angiographic findings excluded embolism and dissection as the cause of the MCA stenosis. Our considerations for the differential diagnosis of this unusual finding focused on the presence of vasculitis or reversible vasoconstriction syndrome. The generalized caliber reduction of the pial vessels and the morphology of the left M1/M2 bifurcation stenosis made any mechanical procedure (balloon dilatation, stent implantation) appear inadequate and unacceptably risky. We performed intra-arterial spasmolysis and slowly administered a dose of 6 mg milrinone into the left ICA over 30 min. The subsequent DSA confirmed an improvement of the stenosis in the left MCA with better perfusion in the distal branches.

After the endovascular procedure the patient remained intubated. Broad-spectrum antibacterial therapy with 2 × 375 mg clarithromycin IV daily and antiviral therapy with 3 × 150 mg acyclovir IV daily were initiated while awaiting a definitive diagnosis. IV nimodipine (2 mg/h) and antiplatelet therapy with 100 mg acetylsalicylic acid (ASA) per os (PO) daily and low molecular weight heparin (LMWH; 400 IU/KG/d) were also initiated. On day 2 after clinical onset, LMWH was switched to daily subcutaneous (SC) administration of 20 mg enoxaparin.

A cerebrospinal fluid (CSF) sample on day 1 showed pleocytosis (39 leukocytes/μl), elevated lactate (5 mmol/l), high protein (194 mg/dl), and a positive anti-Borrelia antibody index for IgG (7.9 for a normal value < 1.5) and IgM (2.1 for a normal value < 1.5) consistent with LNB. On serology studies, anti-Borrelia burgdorferi IgG (> 240 for a normal value < 10) and IgM (54.5 for a normal value < 18) antibodies were demonstrated in the serum. Based on the other laboratory findings, there was no indication of any other type of vasculitis or tuberculosis. Polymerase chain reaction (PCR) was negative for common viruses. On day 1 after clinical onset, the patient was started on antibiotic therapy with 3 × 2 g ceftriaxone IV daily for 5 days and supported with three pulses of intravenous high-dose steroids (methylprednisone, 800 mg (17 mg/kg) for each pulse) on days 1 to 3.

**Fig. 3:**
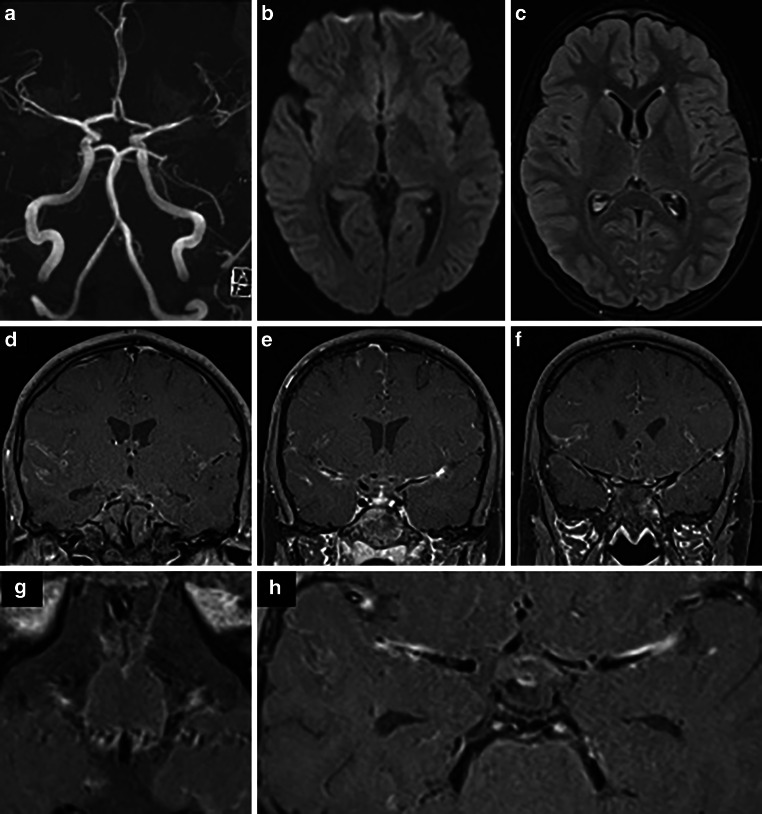
MRI of a 13-year-old boy obtained 1 day after the clinical onset of cerebral vasculitis due to LNB. MIP images of TOF angiography (**a**) demonstrated better arterial flow in the left MCA branches. DWI (**b** = 1000 s/mm2) (axial view; **b**) and FLAIR images (axial view; **c**) showed a decrease in ischemic changes within the left insular cortex. Fat-saturated contrast-enhanced T1-weighted images (coronal views: **d**, **e**, **f**; axial views: **g**, **h**) revealed diffuse leptomeningeal enhancement and arterial wall thickening with contrast enhancement in the both MCA and basilar artery

On day 1 after clinical onset, an MRI with vessel wall imaging (VWI) showed diffuse meningeal and vessel wall contrast enhancement, consistent with meningitis and multifocal cerebral vasculitis involving medium and large vessels in both anterior and posterior circulation, predominantly the left MCA (Fig. 3). No new cerebral ischemic lesion was detected.

After extubation, both the National Institutes of Health Stroke Scale (NIHSS) score and the modified Rankin Scale (mRS) score were 0. The patient underwent daily transcranial Doppler ultrasound examinations, which showed no worsening of the stenosis. Nimodipine was given orally from day 15 (60 mg every 6 h) and then gradually tapered. From day 1 to day 5, 3 × 2 g ceftriaxone was administered IV daily and switched to oral administration (2 g/d) for 2 weeks. Methylprednisolone was switched to IV prednisone (100 mg/d) until day 14, followed by a transition to oral administration with a gradual tapering scheme. Gastric protection commenced with oral administration of 40 mg pantoprazole daily during steroid treatment.

**Fig. 4:**
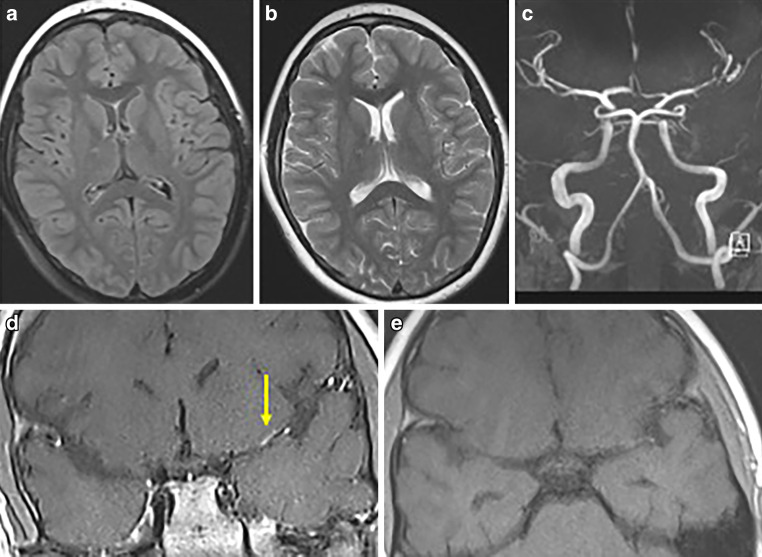
Three-month follow-up MRI after clinical onset and subsequent treatment of cerebral vasculitis due to LNB. FLAIR image (axial view; **a**) and T2-weighted image (axial view; **b**) showed no new cerebral lesions. MIP images from TOF angiography (**c**) demonstrated residual non-flow-limiting stenosis at the left MCA bifurcation. Fat-saturated contrast-enhanced T1-weighted images (coronal view, **d**; axial view, **e**) detected residual minimal vessel wall enhancement of the left distal M1 segment (yellow arrow, **d**) and no leptomeningeal enhancement

At the 3‑month follow-up, the patient had no neurological deficit. A subsequent MRI scan showed residual mural enhancement in the left distal M1 segment with no significant stenosis and excluded the presence of new cerebral lesions (Fig. 4). No new clinical symptoms were observed in 1‑year follow-up.

### Patient 2

**Fig. 5:**
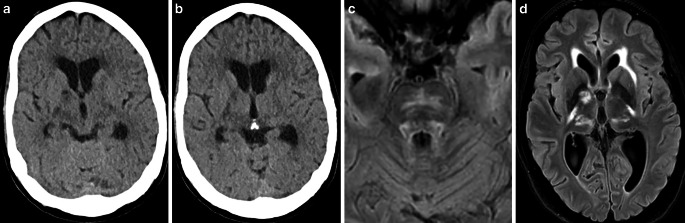
CT and MRI scans of a 57-year-old female presenting with confusion, speech disorder, bradykinesia, and mild right ptosis. Native CT images (axial views; **a**, **b**) and MRI with FLAIR images (axial views; **c**, **d**) demonstrated new lesions in the bilateral thalamus, bilateral internal capsule, left external capsule, and pons

A 57-year-old female patient reported lethargy, vomiting with weight loss, gait disturbance, and rotational dizziness over the 5 months before her recent hospital admission. Despite undergoing a clinical examination, a head computed tomography (CT) scan, and a cervical spine MRI, no apparent cause for her symptoms could be identified. About 3 months later, a neurological examination showed gait ataxia and saccadic pursuit eye movements, and a subsequent electroencephalogram (EEG) revealed generalized slowing. One week following the last clinical examination, the patient presented to our emergency department with an abrupt onset of speech disorder, bradykinesia, mild right ptosis, and confusion. On admission, brain imaging revealed new focal lesions in the bilateral thalamus, bilateral internal capsule, and pons, with no evidence of acute cerebral ischemia (Fig. 5).

**Fig. 6:**
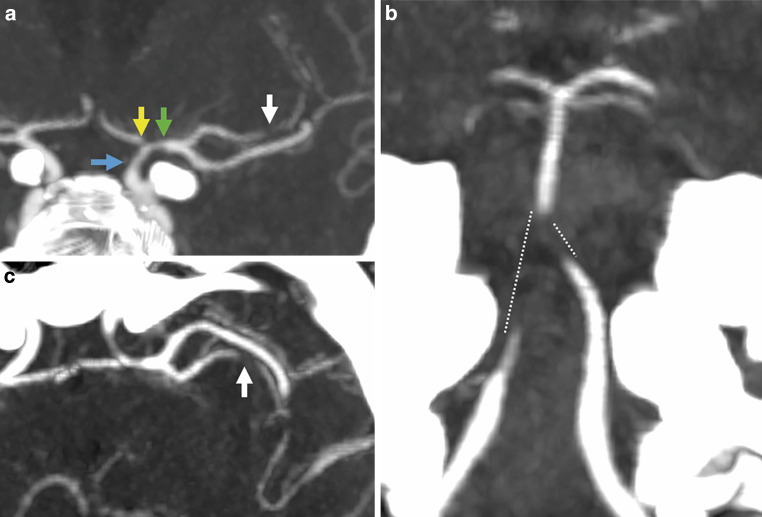
CTA of the same patient showing stenoses of the left distal ICA (**a**, blue arrow), the left proximal A1 segment (**a**, yellow arrow), the left M1 segment (**a**, green arrow), the left M2 segment (**a**, **c**, white arrows), both distal V4 segments, the proximal basilar artery (**b**, dotted lines)

CT angiography (CTA) revealed stenotic changes in intracranial arteries, predominantly in the superior trunk of the left MCA und in the distal V4 segment of the both VA (Fig. 6).

**Fig. 7:**
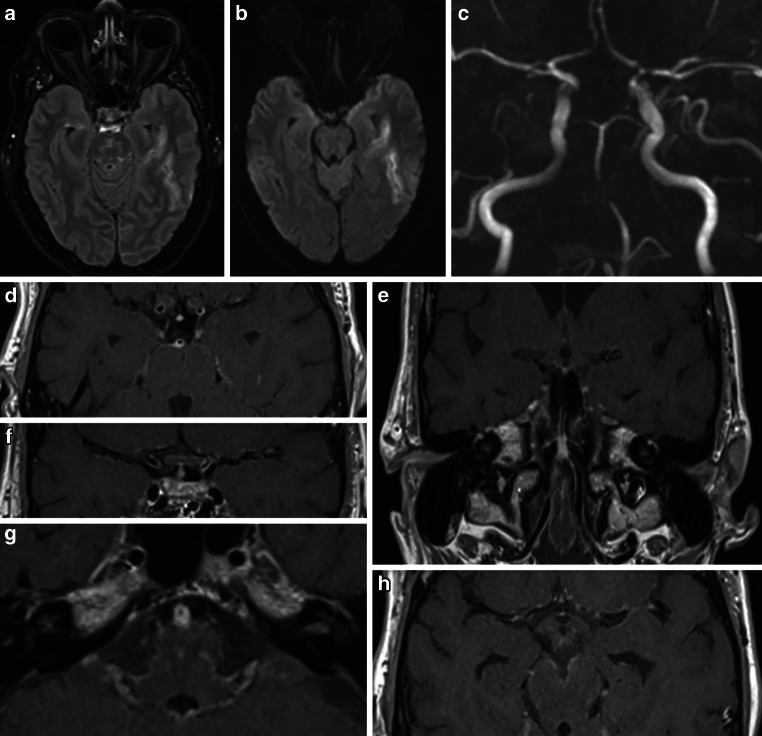
MRI of a 57-year-old patient in the early acute phase of neuroborreliosis-induced vasculitis on day 1. FLAIR (axial view; **a**) and DWI image (axial view; **b**) demonstrating new ischemic area in the left temporal lobe. MIP of the TOF angiography (**c**) confirming previously seen intracranial stenoses (distal left ICA, left ACA/A1, left MCA—M1 and superior trunk, bilateral vertebral artery/V4, proximal basilar artery, bilateral PCA/P2). Fat-saturated post-contrast T1-weighted images (axial views—**d**, **f**, **g**, **h**; coronal view—**e**) showing mural thickening and contrast enhancement in stenotic vessel segments, and basal leptomeningeal contrast enhancement

Medicinal therapy was initiated, as endovascular interventions were not deemed necessary due to the stable clinical condition. On day 1, MRI with VWI showed presence of the mural thickening and contrast enhancement in the vessel wall of the both distal ICA, both MCA, both ACA, both V4 segments, BA and both PCA, new cortical ischemia in the left temporal lobe, as well as basal leptomeningeal contrast enhancement, indicative of basal meningitis and raising suspicion for neurotuberculosis (Fig. 7).

Anti-tuberculosis drug therapy was initiated but later halted after tuberculosis was ruled out. A CSF sample on day 1 showed following results (normal values in brackets): glucose 20 mg/dl [40–75], lactate 3.6 mmol/L [1.2–2.2], total protein 280 mg/dl [15–55], < 1000 erythrocytes/μl [< 1000], 316 leukocytes/μl [0–5]; 10.6 ASI-*B burgdorferi* IgG in CSF [< 1.5], and 6.5 ASI-*B burgdorferi* IgM in CSF [< 1.5]. In the serological examination *B burgdorferi* IgG was > 240, and IgM was 4.5. Other laboratory findings ruled out any alternative infections or vasculitis. The patient was started on antibiotic therapy with 2 × 2 g ceftriaxone IV daily and supported with IV steroids. Gastric protection with 1 × 40 mg pantoprazole PO daily was initiated during steroid treatment.

**Fig. 8:**
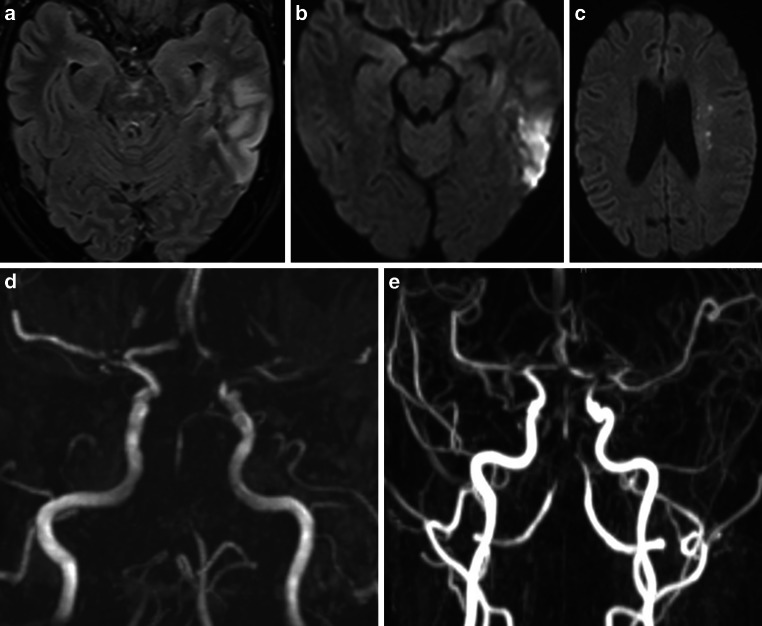
MRI of a patient with severe LNB-associated cerebral vasculitis on day 11 after clinical deterioration. FLAIR image (axial view; **a**) and DWI images (axial views; **b**, **c**) showed progressed left temporal lobe infarction and new ischemic lesions in the deep white matter (watershed zone, hemodynamic infarcts). MIP of the TOF (**d**) and contrast-enhanced MR angiography (**e**) demonstrated worsening of the known intracranial stenoses

On day 11, the patient’s clinical condition deteriorated. She exhibited moderate right-sided hemiparesis and fluctuating aphasia. Progressive ischemic changes were noted in the left temporal lobe, along with new focal ischemic foci in the left semioval center, while stenotic changes in intracranial arteries deteriorated (Fig. 8).

**Fig. 9:**
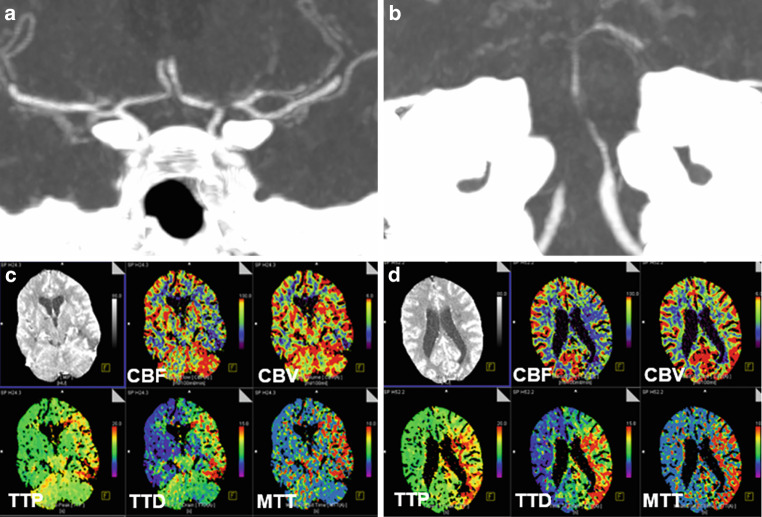
CTA and CTP obtained on day 14 after the onset of right hemiplegia and altered consciousness of a patient with LNB-associated severe cerebral vasculitis. CTA images (coronal views; **a**, **b**) showing critical stenosis of the left ACA/A1, left MCA/M1/superior trunk, bilateral vertebral artery/V4, and the BA. The time-to-drain (TTD) and mean transit time (MTT), determined by CTP (**c**, **d**), is prolonged in the entire posterior circulation and the left anterior circulation with concomitant cerebral blood volume (CBV)-cerebral blood flow (CBF) mismatch

After interdisciplinary discussion, the patient was kept under conservative management with antibiotics, steroids, oral antiplatelet therapy with 1 × 100 mg ASA, and nimodipine IV. On day 14, the patient further deteriorated with right-sided hemiplegia, global aphasia, leftward gaze deviation, and stupor (NIHSS score of 22). CT scan with CT perfusion (CTP) and CTA showed known high-grade intracranial stenoses with reduced perfusion in the left anterior circulation and throughout the posterior circulation (Fig. 9).

The patient was critically at risk due to the rapidly progressing stenoses of the distal VA, BA, and left distal ICA, and left A1 segment of the ACA, as well as M1 and superior trunk of the left MCA. Relying solely on medication-based treatment appeared inadequate for addressing the hemodynamic compromise affecting both posterior and left anterior circulation. Unlike atherosclerotic stenoses, inflammatory-induced stenoses are associated with extreme vulnerability of the vessel wall. Balloon angioplasty with even a slightly undersized balloon and minimal inflation pressure might result in dissection or rupture of the stenotic vessel. Moreover, active mural inflammation may lead to early restenosis. Thus, balloon dilation or the implantation of a balloon-expandable stent was not a preferred option. On the other hand, implantation of a self-expanding stent with minimal radial force (e.g., Neuroform Atlas, Stryker) might not achieve sufficient vessel dilation. A self-expanding stent with sufficient radial force such as the Solitaire stent (Medtronic) had the potential to enhance perfusion. Aside from the relatively high radial force, the Solitaire stent could be tentatively deployed and its effect on vessel dilation could be evaluated with DSA.

**Fig. 10:**
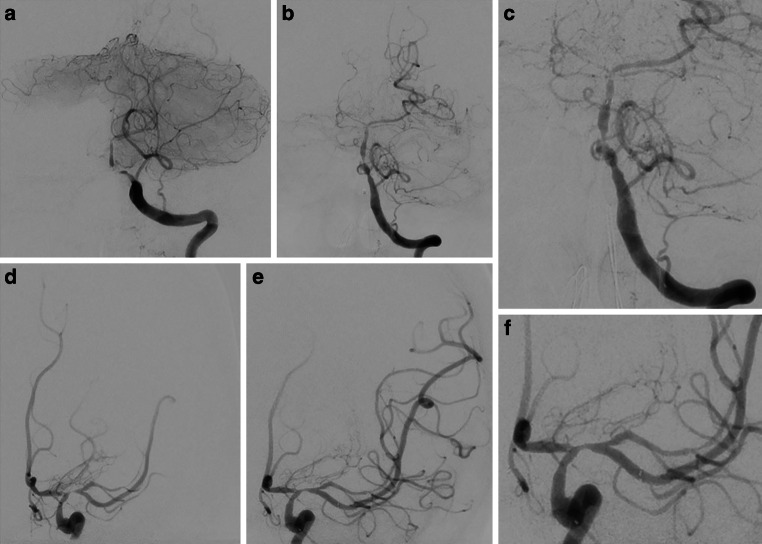
Endovascular treatment of the severe intracranial stenoses in a patient with severe LNB-associated cerebral vasculitis. Diagnostic angiograms of the left VA (PA view; **a**) and the left ICA (PA view; **d**) demonstrating severe stenosis of the left VA-BA junction and the left proximal M1 segment. Angiograms after the stent implantation in the left VA-BA junction (Solitaire 3/20 mm; PA views – **b**, **c**) and in the left M1 segment (Solitaire 4/20 mm; PA views – **e**, **f**) showing the marked improvement of the antegrade arterial flow with residual stenosis

A loading dose of 1 × 180 mg ticagrelor PO was administered. Antiplatelet tests revealed adequate response to the dual antiplatelet therapy. We performed then a bailout stenting with Solitaire-Stent in the left VA-BA junction, and in the left M1 segment. The vessels were angiographically visualized, and stents was then electrolytically detached. Vessel dilatation corresponded to a marked improvement in perfusion of the left anterior and posterior circulations (Fig. 10).

CT scan performed 2 days after the endovascular procedure revealed no new cerebral ischemia or intracranial hemorrhage. The untreated stenoses of the right V4, left A1 segment, and superior trunk of the left MCA remained unchanged from previous imaging evaluations. No new clinical or neurological deterioration on further hospital course was observed. On the day 26, the patient was transferred to a rehabilitation clinic with moderate right hemiparesis and aphasia (NIHSS score of 6). She was maintained on dual antiplatelet therapy with of 2 × 90 mg ticagrelor PO daily for 12 months and 1 × 100 mg ASA PO daily for lifelong.

**Fig. 11:**
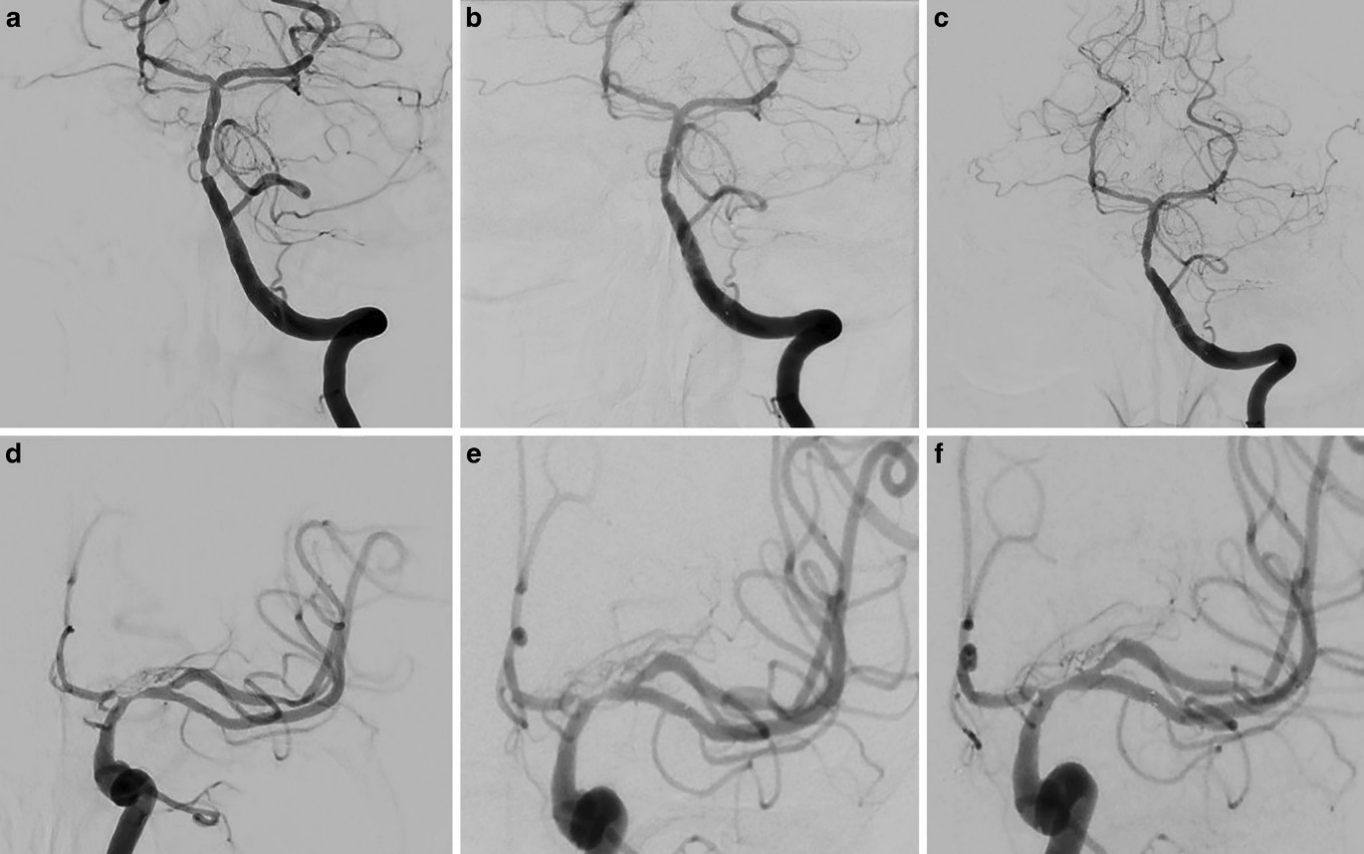
Follow-up DSA examinations after stenting for critical progressive intracranial stenoses due to LNB-related cerebral vasculitis. Angiograms of the left VA (PA views; **a**, **b**, **c**) and the left ICA (PA views; **d**, **e**, **f**) after 3, 6 and 12 months demonstrating a positive remodeling effect of the stent, with no significant residual stenosis

**Fig. 12:**
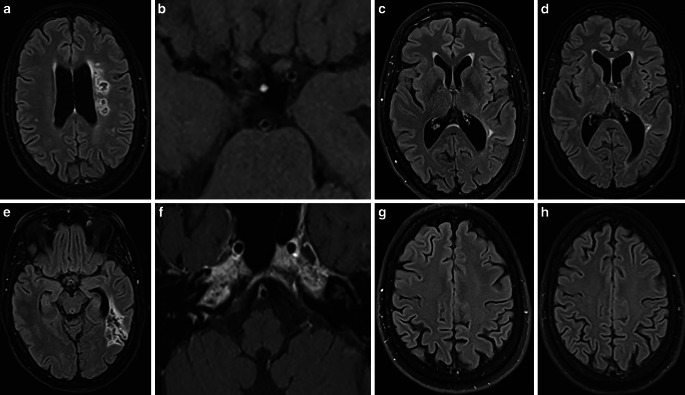
Follow-up MRI scans after stent angioplasty for intracranial stenoses secondary to a LNB-associated cerebral vasculitis. FLAIR images (axial views; **a**, **e**) and fat-saturated contrast-enhanced T1-weighted images (axial views; **b**, **f**) after 3 months demonstrating unchanged chronic infarct areas in the left temporal lobe and in the left semioval center, and regressed mural thickening and contrast enhancement in the intracranial arteries. FLAIR images after 6 months (axial views; **c**, **g**) and 12 months (axial views; **d**, **h**) showed no new cerebral lesions

DSA examinations conducted at 3, 6, and 12 months after stent angioplasty demonstrated an improvement in stenosis and a positive remodeling effect of the stent in the BA and the left M1 segment (Fig. 11). Follow-up MRI scans showed no new cerebral lesions and decreased leptomeningeal and mural contrast enhancement (Fig. 12). At 1‑year follow-up she had significant clinical improvement and reached an mRS score of 1 (mild speech disorder and reduced fine motor skills of the right hand).

### Patient 3

**Fig. 13:**
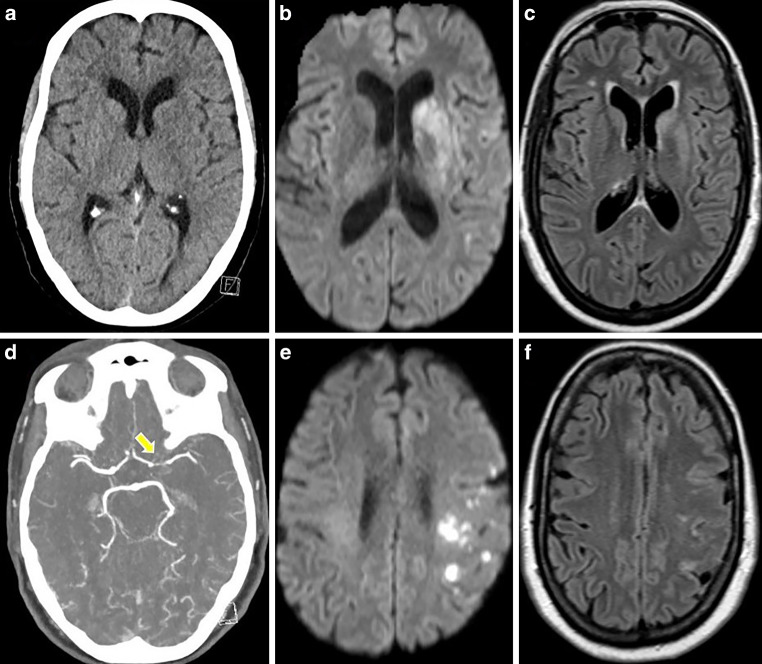
CT and MRI scans of a 56-year-old female presenting with right hemiparesis and a speech disorder. Native CT scan (axial view; **a**) showed a suspicious ischemic lesion in the left basal ganglia. DWI images (b = 1000 s/mm2, axial views; **b**, **e**) demonstrated acute ischemia in the left basal ganglia and the left pre- and post-central areas with correlation in FLAIR images (axial views; **c**, **f**). CT angiography (axial view; **d**) revealed high-grade stenosis in the left proximal M1 segment (yellow arrow)

A 56-year-old female patient initially presented to a peripheral hospital with mild right hemiparesis and a slight speech disorder. Initial CT scan showed a suspicious ischemic lesion in the left basal ganglia and a high-grade proximal left M1 stenosis (Fig. 13). Within four hours, her clinical condition worsened, and subsequent MRI revealed acute ischemia in the left basal ganglia and multiple ischemic foci in the left pre- and post-central areas (Fig. 13).

Due to her deteriorating condition, the patient was transferred to our clinic. On admission, she had a right hemiplegia, right-sided facial nerve palsy, global aphasia, and disorientation (NIHSS score 13). About a week earlier, she was admitted to another hospital for headaches, fatigue and recurrent word-finding difficulties for several weeks, but no abnormalities were found on the CT scan. While obtaining the patient’s medical history with her daughter, it was revealed that she had a history of a tick bite and an erythema migrans approximately 6 months earlier, which was not treated.

**Fig. 14:**
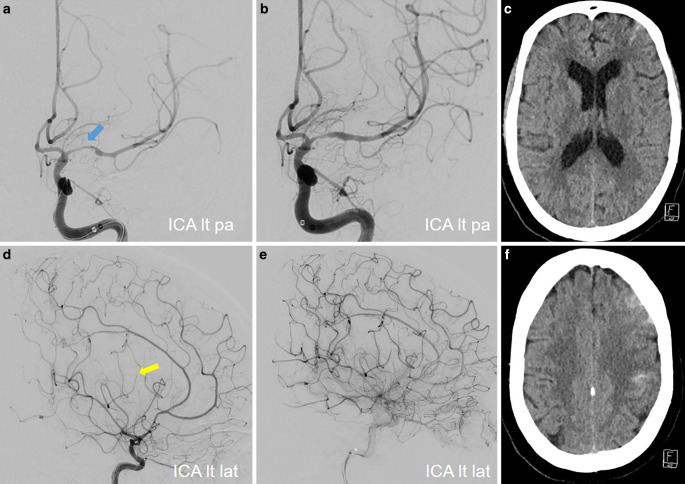
Endovascular treatment of the severe intracranial stenosis with non-occlusive thrombus in the proximal left M1 segment. Diagnostic angiograms of the left ICA (PA view, **a**; lateral view, **d**) demonstrating severe stenosis of the left M1 segment with adjacent thrombus (blue arrow), distal perfusion delay and a peripheral embolus in a precentral branch (yellow arrow). Angiograms after stent implantation (PA view, **b**; lateral view, **e**) showed complete coverage of the lesion with an improved distal perfusion. Native CT scan on day 1 after treatment (axial views; **c**, **f**) demonstrated minimal SAH in the sulci of the left frontal and parietal lobes and no new cerebral infarction

Upon arrival at our clinic, the patient was promptly taken to the neuroangiography suite. Angiograms of the left ICA revealed a high-grade long-segment stenosis in the proximal left M1 with adjacent thrombus formation, a peripheral embolus in a precentral branch, and reduced perfusion in the distal MCA branches (Fig. 14). No hemodynamically significant focal stenosis was observed in the posterior and right anterior circulation. In the context of acute ischemic stroke with severe stenosis and thrombus, initially unknown origin, we have opted for endovascular treatment over conservative management alone. Nevertheless, the long segment involvement was suspicious for possibly other etiology as classic atherosclerosis. We decided therefore against balloon dilatation or mechanical thrombectomy and instead opted for implantation of a self-expandable stent (Solitaire, Medtronic). A loading dose of 1 × 180 mg ticagrelor PO, 1 × 500 mg ASA IV and 1 × 14.6 mg eptifibatide IV was administered. After stent deployment the vessels were visualized angiographically, and then the stent was electrolytically detached. In the control angiogram, complete coverage of the lesion with a notable enhancement in perfusion of the left anterior circulation was observed (Fig. 14). CT scan on the day 1 demonstrated known subacute ischemia and mild subarachnoid hemorrhage (SAH) in the left frontal and parietal sulci (Fig. 14).

**Fig. 15:**
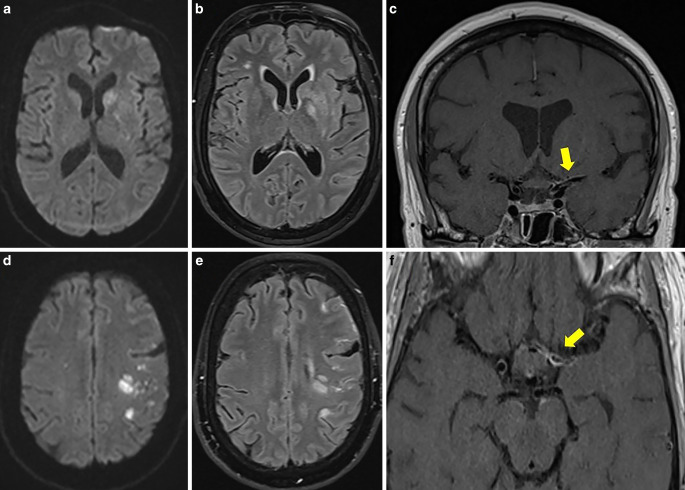
MRI examination after stenting of the left MCA in a patient with severe left M1 stenosis due to LNB-associated vasculitis. DWI images (b = 1000 s/mm2, axial views; **a**, **d**) and FLAIR images (axial views; **b**, **e**) demonstrated known ischemic lesions in the left basal ganglia and the left frontoparietal lobe, and residual sulcal SAH. Fat-saturated post-contrast T1-weighted images (coronal view—**c**; axial view—**f**) showing mural contrast enhancement in the left M1 segment and proximal A1 segment

The patient was then successfully extubated. A CSF sample on day 1 showed pleocytosis (340 leukocytes/μl), elevated lactate (5.3 mmol/l), decreased glucose (14 mg/dl), high protein (76.3 mg/dl), and a positive anti-Borrelia antibody index for IgG (22.5 for a normal value < 1.5) and IgM (9.1 for a normal value < 1.5) consistent. On serology studies, anti-Borrelia burgdorferi IgG (98.3 for a normal value < 10) and IgM (62.1 for a normal value < 18) antibodies were demonstrated. Other laboratory findings ruled out any alternative infections or vasculitis. She was treated with 2 × 2 g ceftriaxone IV daily and dual antiplatelet therapy with 2 × 90 mg ticagrelor PO daily, 1 × 100 mg ASA PO daily. On the day 2, MRI with VWI revealed arterial vessel wall thickening and contrast enhancement in the distal ICA, in both A1 and M1 segments, in the distal V4 segments and in the BA without significant flow reduction. No new cerebral ischemia was detected, and SAH was almost completely reabsorbed (Fig. 15).

**Fig. 16:**
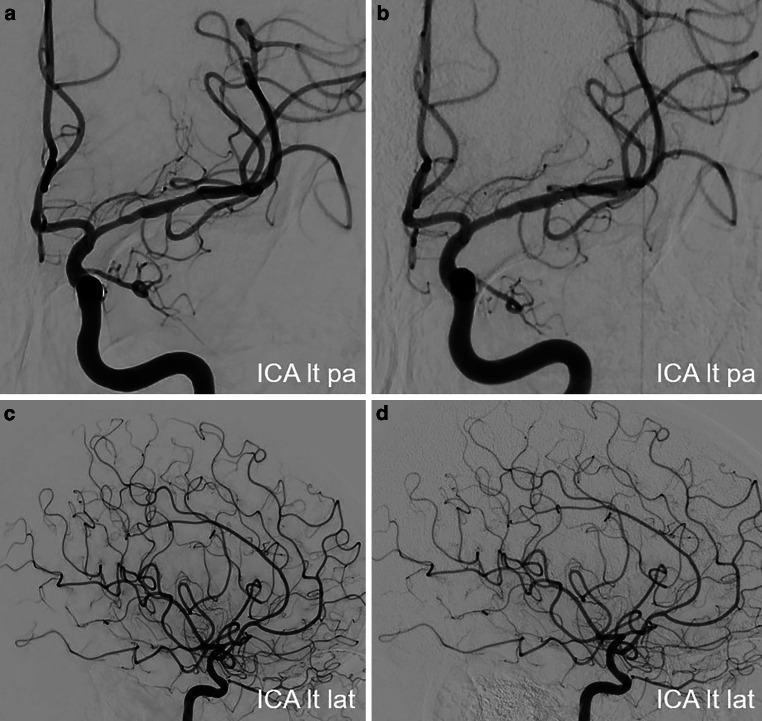
Follow-up DSA examinations after stenting for severe left M1 stenosis due to LNB-related vasculitis. Angiograms of the left ICA after 6 (PA views; **a**, **c**) and 12 months (PA views; **b**, **d**) demonstrating a positive remodeling effect of the stent, with no significant residual stenosis

No new neurological events were reported, and her clinical condition continued to improve in further hospital course. Upon discharge on the day 6, her mRS score was 3 and NIHSS score was 7. The follow-up DSA examinations in 6 and 12 months demonstrated the stent patency, with no in-stent restenosis (Fig. 16). She showed significant clinical improvement in 1‑year follow-up period to mRS 1 (mild reduced fine motor skills of the right hand).

## Discussion and Literature Review

Neurological symptoms typically manifest within 1–12 weeks (mostly 4–6 weeks) after a tick bite by *Ixodes ricinus*, transmitting the spirochete *Borrelia burgdorferi sensu latu*, primarily from July to December. Early LNB, accounting for over 95% of cases, is characterized by signs and symptoms lasting less than 6 months, while less than 5% represent late LNB, extending from 6 months to several years. Although LNB commonly presents with facial nerve palsy, meningitis, and radiculopathy, differences in clinical presentation between European and American populations likely arise from variations in spirochete species [2]. The range of clinical manifestations also frequently corresponds to the age of the patient. Children commonly exhibit facial nerve palsy, lymphocytic meningitis accompanied by subacute headache, moderate neck stiffness, and low-grade fever, while painful meningoradiculitis is more prevalent among adults [8]. While the absence of a history of tick bites or EM is common [9], non-specific symptoms should not lead to dismiss the possibility of lyme disease. In suspected cases of LNB, a lumbar puncture is often performed as part of the diagnostic protocol [2]. CSF analyses typically reveal lymphocytic pleocytosis, elevated protein and lactate levels, and decreased glucose levels [10]. The detection of intrathecal synthesis of antibodies against *Borrelia burgdorferi* confirms the diagnosis of definite LNB [2].

In rare cases, LNB can also have a complicating course, including cerebral vasculitis with severe stenosis or occlusion and consequent cerebral ischemia [5]. The estimated incidence of cerebral vascular complications of LNB in endemic areas is 0.3–1% [11, 12]. Clinically, although manifestations are variable, signs of posterior circulation stroke or cerebellar dysfunction are particularly common [11]. A review study of 88 individual cases [6] reported that the most common cerebrovascular manifestation of LNB was ischemic stroke (76.1%), followed by TIA (11.4%). The posterior cerebral circulation was predominantly affected (37.8%), while 20 patients (24.4%) exhibited involvement of the anterior circulation, and both anterior and posterior circulation were implicated in 31 cases (37.8%).

MRI with VWI is the preferred imaging modality for early detection of cerebrovascular manifestations of LNB, given its frequent association with cerebral vasculitis [5, 6]. Such cerebrovascular manifestations of LNB in the pediatric population are exceptional but deserve special attention to allow prompt initiation of therapy [13].

Effective treatment of neuroborreliosis requires antibiotic therapy, but initiation of these medications depends on a timely diagnosis of LNB, which is often delayed or missed due to the presentation of non-specific symptoms. According to the last published guidelines [4], patients with early LNB should be treated with antibiotics for 14 days and late LNB for 14–21 days. In cases of cerebral vasculitis, antibiotics should be administered in accordance with the recommendations for late LNB.

Our analysis of the available literature showed limited reports of adjunctive neurointerventional therapies in LNB-associated significant large-vessel inflammatory stenoses or occlusions. The safety and effectiveness of reperfusion therapies in cerebral vasculitis with infectious and inflammatory components are largely unknown.

In one case report, stent-retriever thrombectomy were performed in a 6-year-old child with acute basilar artery occlusion due to LNB. In this case, rapid diagnosis, and timely endovascular intervention, along with antibiotic and anti-inflammatory treatment, resulted in an excellent outcome [14]. In another case, a 52-year-old woman with cerebral ischemia and hemodynamically relevant right MCA stenosis due to LNB-associated vasculitis was treated with repeated IA spasmolysis, balloon angioplasty, and stenting. The authors noted a short-term neurological improvement after endovascular intervention, but the vasculitis worsened during the hospital course and the occurrence of intracranial hemorrhage contributed to a poor outcome, resulting in death [15]. Vandelli et al. [16] documented two cases involving suspected cerebral vasculitis with severe intracranial stenosis and acute stroke, where standard medical treatment had limited efficacy. Due to clinical deterioration and hemodynamic instability, these patients underwent endovascular treatment with stenting, resulting in remarkable clinical improvement.

Inflamed, stenotic vessels are believed to be fragile, risking dissection or rupture during balloon dilation. Reports from cardiovascular studies shows that stent implantation in inflamed vessels might trigger a foreign body response, leading to higher rates of an in-stent restenosis [17]. On the other hand, severe and extensive inflammatory stenoses, as seen in the second patient presented here, poses an immediate life-threatening and arguably insurmountable challenge. We believe that in the absence of therapeutic alternatives and for critically endangered patients, the implantation of a self-expanding stent as a bailout strategy may offer a potential solution to alleviate inflammatory-induced intracranial stenosis of medium to large arteries. Persisting with a passive approach in the presence of severe clinical symptoms and cerebral ischemia might have led to an unfavorable clinical outcome due to the expected occurrence of further ischemic events.

## Conclusion

The diagnosis of LNB is challenging and requires great clinical acumen and awareness due to the variety of non-specific symptoms that often lead to potential misdiagnosis and delays in treatment. Endovascular interventions as a bailout strategy may enhance clinical outcomes in endangered patients with severe vascular complications of LNB, especially when medicinal therapy alone fails to yield further improvement. In the setting of severe ischemic stroke with sub-occlusive large vessel stenosis or occlusion, the cause of which is often unknown, it should be considered to prioritize prompt endovascular treatment, even if LNB is suspected on admission.
